# Prevalence of Chagas Disease Among Family Members of Previously Diagnosed Patients in Los Angeles, California

**DOI:** 10.1093/cid/ciz087

**Published:** 2019-03-28

**Authors:** Salvador Hernandez, Colin J Forsyth, Carmen A Flores, Sheba K Meymandi

**Affiliations:** Center of Excellence for Chagas Disease at Olive View-University of California, Los Angeles Medical Center, Sylmar

**Keywords:** Chagas disease, *Trypanosoma cruzi*, screening, neglected diseases

## Abstract

Chagas disease (CD) in the United States is severely underdiagnosed, due to an absence of systematic screening as part of routine healthcare. We screened 189 relatives of 86 existing patients and found a CD prevalence of 7.4%. Screening close relatives of previously diagnosed individuals can effectively identify new CD cases.

Chagas disease (CD), caused by the protozoan *Trypanosoma cruzi*, affects >6 million people globally, including over 300 000 in the United States [1, 2]. It causes a greater burden in terms of disability-adjusted life-years than any other parasitic disease in the Americas, where it is a leading cause of heart failure [3]. Timely treatment with antitrypanosomal drugs improves morbidity and mortality from CD and eliminates the potential for congenital transmission [4, 5]. Such treatment must be administered while patients are still in the asymptomatic, indeterminate phase of the disease, when they are likely to be unaware of the infection, underscoring the importance of systematic screening programs. However, <1% of people with the disease in the United States have received diagnosis and etiologic treatment [6], highlighting an urgent need to implement effective interventions for screening and detecting cases.

In the United States, CD is concentrated among people born in Latin America, even though local transmission and infection is periodically documented. Blood donations have been screened since 2007; however, routine screening does not occur within primary care centers or during pre- and postnatal care, and many US physicians are unfamiliar with CD [6]. The World Health Organization recommends screening relatives of *T. cruzi-*infected individuals. Congenital transmission affects the distribution of CD within families; in addition, relatives, including siblings and spouses, are likely to share exposures and risk factors [7], resulting in an elevated risk of CD. In this investigation, we assess the prevalence of CD among spouses, siblings, parents, and children of *T. cruzi-*infected individuals in Los Angeles, California.

## METHODS

We assessed the prevalence of *T. cruzi* infection in a convenience sample of 189 relatives of 86 patients with CD who had previously been identified at the Center of Excellence for CD (CECD) at the Olive View-University of California, Los Angeles (UCLA) Medical Center, a safety-net facility in Sylmar, California, serving Los Angeles County. The original 86 patients with CD were identified through the CECD’s community-based screening program or referrals from blood banks or other providers in Los Angeles. They represent a subset of the CECD’s total patient population, consisting of those who had close relatives in the Los Angeles area and consented to participate in this investigation. During the study period (August 2007 through February 2017), these patients were asked to bring in close relatives for testing, and through this process 189 relatives were recruited and tested. The study was approved by the Institutional Review Board of the Education and Research Institute at Olive View-UCLA Medical Center, and all participants underwent an informed consent process.

Blood samples were initially tested for *T. cruzi* antibodies using a Chagatest enzyme-linked immunosorbent assay v.3.0, Wiener Lab Group, Rosario, Argentina (sensitivity = 98.8%, specificity = 99.6%) [8]. Positive results were then confirmed at the Centers for Disease Control with an immunofluorescence antibody assay or an immunoblot assay using trypomastigote excreted-secreted antigens (sensitivity and specificity = 100%) [9]. Respondents were offered an electrocardiogram. We also collected data on participants’ age, countries of origin, and length of time both in the United States and, for those born abroad, in their home countries. These data were analyzed using Mann-Whitney *U* tests for continuous variables and χ^2^ or Fisher exact tests (if cell sizes were <5) for categorical variables. Statistical analysis was performed using SPSS v. 24.0 (IBM, Armonk, New York).

## RESULTS

The 189 family members tested ranged in age from newborn to 73 years old; 59.3% were female, and 33.9% were children younger than 18 years old (Table 1). Nearly half of the respondents (n = 86) were born in the United States; others came from El Salvador (n = 55), Mexico (n = 24), and elsewhere in Latin America. The majority (138, 73.0%) had a parent with CD, either a mother (n = 114) or a father (n = 24). The remaining participants were siblings (n = 28), spouses (n = 16), parents (n = 5), or other relations (son-in-law = 1, niece = 1).

**Table 1.** Prevalence of *Trypanosoma cruzi* Infection and Comparison of Seropositive and Seronegative Groups

Category	*Trypanosoma cruzi* +	*Trypanosoma cruzi* −	Prevalence	*P* Value
All	14	175	7.4	
Females	9	103	8.0	.691
Males	5	72	6.5	
Children <18	1	63	1.6	.037
Adults >18	13	112	10.4	
Adults >40	10	29	25.6	<.001
Birth country				
U**nited** S**tates**	1	85	1.2	.004
El Salvador	9	46	16.4	.005
Mexico	2	22	8.3	.693
Other	2	22	8.3	.693
Relationship to case^a^				
Maternal child	4	110	3.5	.020
Paternal child	0	24	0.0	.222
Sibling	7	21	25.0	.001
Spouse	1	15	6.3	.853
Parent	2	3	40.0	.045
Electrocardiogram results				
Normal	5	124	7.1	.095
Conduction abnormality	2	9	27.3	
Mean age, y	47.8	24.4		<.001
Mean years in the U**nited** S**tates**	23.7	17.0		.022
Mean years in country of origin	31.9	13.1		<.001

^a^Others were a niece and son-in-law.

A positive CD diagnosis was confirmed in 14 participants, resulting in an overall prevalence of 7.4%. There was a substantial gap in mean age between *T. cruzi* positive (47.8) and negative (24.4) individuals, and prevalence was much higher in adults than children. Further, 10/14 seropositive individuals (71.4%) were over 40. Based on birth countries, El Salvador had the highest prevalence (9/55, 16.4%, *P* = .005). All 14 participants with CD were offered treatment at the CECD; 6 were treated and the remainder declined (n = 4) or were lost to follow-up (n = 4).

Siblings (7/28, 25%, *P* = .001) and parents (2/5, 40%, *P* < .045) had the highest prevalence of any relationship category. Among all 138 participants who had a parent with CD, only 4 (2.9%) were seropositive; all were maternal offspring. Only one of 64 children under 18 had a positive diagnosis (a probable but unconfirmed congenital case of a 2-month old infant born to a Salvadoran mother); this was also the only case detected among US-born participants.

Electrocardiogram results were available for 140 participants. Two of 11 seropositive respondents exhibited abnormalities on electrocardiogram, compared with 5/129 seronegative respondents. One seropositive respondent displayed a right bundle branch block, whereas the other exhibited sinus bradycardia.

## CONCLUSIONS

Although congenital transmission of CD has been well documented, other epidemiological research on relatives of index cases diagnosed with CD has been limited. This may reflect the impact of regional programs focused on halting congenital transmission, and the fact that treatment recommendations have been largely restricted to children until recently. The few available studies indicate family members of patients represent a high-risk group. A study in Chile began with 70 index cases, all women assessed with *T. cruzi* infection during prenatal care, and tested 349 family members (children, mothers, and siblings). Of the relatives, 148 were confirmed positive (42.4%) [10]. Similarly, 9.3% of relatives of infected mothers were positive for *T. cruzi* in a Colombian study [11]. An Argentinian study where infants born to seropositive mothers were considered index cases determined siblings of infected infants had a high CD prevalence (31%), whereas no CD cases were detected among siblings of noninfected infants [12]. Family members could also represent a higher risk group among immigrant populations, although epidemiological data are limited.

In a large-scale community screening conducted by the CECD of Latin American adults in Los Angeles, the prevalence of *T. cruzi* infection was 1.24% [13]. In the present study of family members of *T. cruzi-*infected individuals, the prevalence among adults (10.4%) was 8 times higher, suggesting relatives are a particularly high risk group. Close relatives share housing, a key risk factor for CD, which likely explains the high prevalence we observed in siblings (25.0). In fact, siblings and parents of index cases had a much higher CD prevalence than maternal children. However, our study relies on a convenience sample, and results are not generalizable to settings outside of Los Angeles. Infected family members who we identified tended to be of older age, but ideally CD should be identified and treated much earlier.

Nonetheless, congenital transmission is an important route of transmission in the United States, accounting for an estimated 63–315 new cases of CD annually [14]. The risk of congenital transmission ranges from 5% in endemic to 2.7% in nonendemic countries [15]. To our knowledge, this is the first US study to screen a large number of children for CD. Out of 64 maternal children under 18, we diagnosed CD in one 2-month old infant born to a seropositive mother, a probable case of congenital transmission, although environmental exposure could not be ruled out. Screening of mothers, newborns, and children, which currently does not take place in the United States, is a highly desirable public health intervention not only for interrupting this route of transmission but because cure rates for antitrypanosmal treatment approach 100% in newborns [7], who also experience a lower rate of adverse reactions to the drugs than older children and adults. Focusing on relatives of *T. cruzi* infected individuals may help identify more children who would benefit from CD screening.

Currently, <1% of the estimated 326 000–347 000 people with CD in the United States [2] are diagnosed and treated; major barriers include low physician awareness, exclusion of vulnerable groups from healthcare, and lack of a standard, straightforward diagnostic procedure [6].

There is a need for dramatic expansion of screening programs, which at present are virtually nonexistent outside of blood banks and organ donations. Our study suggests that when US patients are diagnosed with CD, their close family members are at high risk and should be screened as well. Such family-based screening should be integrated into primary care settings accessible to the population at risk, and this analysis supports the potential value of such an approach. Moreover, our study supports offering screening to relatives of patients who are diagnosed when giving blood or donating/receiving organs, which is currently the main pathway of CD diagnosis in the United States. Detecting *T. cruzi* infection early and proactively, before progression to chronic symptoms, is a crucial step to reducing the heavy burden of CD in the United States and elsewhere.

## Notes

***Acknowledgments.*** The authors thank the patients, staff, and volunteers at the Center of Excellence for Chagas Disease at Olive View-University of California Los Angeles Medical Center for making this study possible. They are grateful to the Parasitic Diseases Branch Laboratory at Centers for Disease Control for support with testing of blood samples, and to Jose Amadeo Flores for help with data searches. They also thank the Drugs for Neglected Diseases *initiative* and Doctors Without Borders/Médecins Sans Frontières USA for their support.

***Potential conflicts of interest.*** C. F. received salary from the Drugs for Neglected Diseases *initiative* and Doctors Without Borders/Médecins Sans Frontières USA while working on this article. All other authors report no potential conflicts. All authors have submitted the ICMJE Form for Disclosure of Potential Conflicts of Interest. Conflicts that the editors consider relevant to the content of the manuscript have been disclosed.
